# Development and validation of a short version of the MD Anderson Symptom Inventory for upper gastrointestinal surgery (short-MDASI-UGI-Surg) for postoperative patient-reported outcome-based care

**DOI:** 10.1093/bjsopen/zrag026

**Published:** 2026-04-15

**Authors:** Taisuke Imamura, Koichi Tomita, Paula Marincola Smith, Maho Takayama, Anneliese Hierl, Xin Shelley Wang, Loretta A Williams, Kyle G Mitchell, Ravi Rajaram, David Rice, Wayne Hofstetter, Mara B Antonoff, Reza Mehran, Ara Vaporciyan, Garrett Walsh, Jessica E Maxwell, Rebecca A Snyder, Michael P Kim, Ching-Wei D Tzeng, Paul Mansfield, Stephen Swisher, Jeffrey E Lee, Brian D Badgwell, Matthew H G Katz, Naruhiko Ikoma

**Affiliations:** Department of Surgical Oncology, The University of Texas MD Anderson Cancer Center, Houston, Texas, USA; Department of Surgical Oncology, The University of Texas MD Anderson Cancer Center, Houston, Texas, USA; Department of Department of Colon & Rectal Surgery, The University of Texas MD Anderson Cancer Center, Houston, Texas, USA; Department of Surgical Oncology, The University of Texas MD Anderson Cancer Center, Houston, Texas, USA; Department of Surgical Oncology, The University of Texas MD Anderson Cancer Center, Houston, Texas, USA; Department of Symptom Research, The University of Texas MD Anderson Cancer Center, Houston, Texas, USA; Department of Symptom Research, The University of Texas MD Anderson Cancer Center, Houston, Texas, USA; Department of Thoracic and Cardiovascular Surgery, The University of Texas MD Anderson Cancer Center, Houston, Texas, USA; Department of Thoracic and Cardiovascular Surgery, The University of Texas MD Anderson Cancer Center, Houston, Texas, USA; Department of Thoracic and Cardiovascular Surgery, The University of Texas MD Anderson Cancer Center, Houston, Texas, USA; Department of Thoracic and Cardiovascular Surgery, The University of Texas MD Anderson Cancer Center, Houston, Texas, USA; Department of Thoracic and Cardiovascular Surgery, The University of Texas MD Anderson Cancer Center, Houston, Texas, USA; Department of Thoracic and Cardiovascular Surgery, The University of Texas MD Anderson Cancer Center, Houston, Texas, USA; Department of Thoracic and Cardiovascular Surgery, The University of Texas MD Anderson Cancer Center, Houston, Texas, USA; Department of Thoracic and Cardiovascular Surgery, The University of Texas MD Anderson Cancer Center, Houston, Texas, USA; Department of Surgical Oncology, The University of Texas MD Anderson Cancer Center, Houston, Texas, USA; Department of Surgical Oncology, The University of Texas MD Anderson Cancer Center, Houston, Texas, USA; Department of Surgical Oncology, The University of Texas MD Anderson Cancer Center, Houston, Texas, USA; Department of Surgical Oncology, The University of Texas MD Anderson Cancer Center, Houston, Texas, USA; Department of Surgical Oncology, The University of Texas MD Anderson Cancer Center, Houston, Texas, USA; Department of Thoracic and Cardiovascular Surgery, The University of Texas MD Anderson Cancer Center, Houston, Texas, USA; Department of Surgical Oncology, The University of Texas MD Anderson Cancer Center, Houston, Texas, USA; Department of Surgical Oncology, The University of Texas MD Anderson Cancer Center, Houston, Texas, USA; Department of Surgical Oncology, The University of Texas MD Anderson Cancer Center, Houston, Texas, USA; Department of Surgical Oncology, The University of Texas MD Anderson Cancer Center, Houston, Texas, USA

**Keywords:** symptom burden, perioperative care, longitudinal assessment, gastrointestinal cancer

## Abstract

**Background:**

The MD Anderson Symptom Inventory for upper gastrointestinal surgery (MDASI-UGI-Surg) is a validated 27-item patient-reported outcome instrument designed to assess perioperative symptom burden and functional status in patients undergoing surgery for upper gastrointestinal cancers. This study aimed to develop a shortened version (Short-MDASI-UGI-Surg) that preserves the sensitivity of the original tool while reducing respondent burden.

**Methods:**

Prospectively collected longitudinal patient-reported outcome data obtained using the MDASI-UGI-Surg or the MDASI in patients with gastrointestinal cancer (MDASI-GI) from patients who underwent oesophagectomy, gastrectomy, or pancreatectomy for cancer were retrospectively analysed. Symptom severity and temporal patterns were examined using hierarchical clustering, and representative symptoms were selected based on clinical relevance and symptom severity through correlation analysis.

**Results:**

Time series clustering of the 27 items collected from 302 patients revealed three distinct symptom/interference clusters based on relative peak timing: acute, with items peaking on postoperative day (POD) 3; subacute, peaking on POD 21; and persistent, extending through postoperative months 3–6. Within each symptom/interference cluster, items were identified that were highly correlated with other items (*r* ≥ 0.55), from which 13 representative symptom or interference items were selected. The 13 selected items were incorporated into the Short-MDASI-UGI-Surg and demonstrated high concurrent validity with the harmonized full MDASI reference (*r* = 0.98) and strong concordance in time series trends across all timepoints (*r* = 0.96–0.98). Known groups validity was supported by significant differences in scores between the oesophagectomy, pancreatectomy, and gastrectomy groups using both the harmonized full MDASI instrument and the Short-MDASI-UGI-Surg.

**Conclusions:**

The 13-item Short-MDASI-UGI-Surg, may reduce respondent burden and enhance clinical implementation while promoting patient engagement in perioperative symptom monitoring.

## Teaser

A 13-item shortened version of the MD Anderson Symptom Inventory for upper gastrointestinal surgery (Short-MDASI-UGI-Surg) was developed and validated using longitudinal patient-reported outcome data from 302 patients undergoing oesophagectomy, gastrectomy, or pancreatectomy. Time series clustering identified acute, subacute, and persistent postoperative symptom patterns, from which representative items were selected based on correlation and clinical relevance. The Short-MDASI-UGI-Surg demonstrated excellent concurrent validity with the full instrument and robust known groups validity, supporting its use for efficient perioperative symptom monitoring.

## Introduction

Patient-reported outcomes (PROs) are essential for evaluating patient symptom burden from disease- and treatment-related toxicity, especially in surgical oncology, where recovery is complex and multifaceted. Unlike traditional metrics such as complication rates or length of hospital stay^1–3^, PROs provide direct insight into the patient experience, capturing physical symptoms, emotional distress, and functional impairments in ways that are both clinically informative and personally meaningful^4–6^. In the perioperative setting, where symptoms can fluctuate dramatically, monitoring symptom burden through PROs is essential for quality improvement, especially for upper gastrointestinal (UGI) cancer surgery, where the physiological insult is profound and the recovery trajectory can vary widely. In this context, monitoring recovery from the patient’s perspective is not only valuable but also necessary to support informed decision-making and optimize patient-centred care.

To address this need, an MD Anderson Symptom Inventory (MDASI) survey^7,8^ was recently developed and validated for patients undergoing UGI cancer surgery (MDASI-UGI-Surg)^9,10^. Although the MDASI-UGI-Surg comprehensively assesses 21 symptom items and six interference items (27 items in total), its implementation in routine clinical practice presents challenges. Although shorter than many commonly used instruments, such as the European Organization for Research and Treatment of Cancer Quality of Life Questionnaire (EORTC QLQ)^11^ and the disease-specific EORTC QLQ-Stomach^12^, together comprising over 50 items, the 27-item length of the MDASI-UGI-Surg still imposes considerable burden for repeated data collection, particularly for patients in the early phases of postoperative recovery. Developing an abbreviated version could make the instrument easier for patients to complete and quicker for clinicians to review while still capturing the most critical information.

Respondent burden also creates logistical challenges for researchers aiming to maintain high response rates. In PRO research, a response rate > 60% is generally considered the lower bound of acceptability^13,14^. However, compliance tends to decrease with longer instruments, especially among patients experiencing severe symptoms. This introduces the risk of reporting bias, where those most affected may be least likely to respond^15,16^. In clinical practice, the primary challenge for longitudinal PRO monitoring arises when certain items are not relevant or occur with very low prevalence in the specific patient cohort, which can limit the usefulness of repeated assessments. A shorter, streamlined survey based on the MDASI-UGI-Surg could reduce respondent burden and enhance clarity while significantly improving clinical usability and data quality, facilitating broader implementation in routine care.

To respond to the need for a streamlined PRO instrument for UGI surgery, a detailed analysis was conducted of PROs collected longitudinally from individuals who underwent oesophagectomy, gastrectomy, or pancreatectomy at The University of Texas MD Anderson Cancer Center. It was hypothesized that, among the 27 symptom or interference items of the MDASI-UGI-Surg, many items demonstrate harmonized temporal patterns and similar severity levels, with strong interitem correlations enabling efficient grouping. On this basis, the aim of this study was to develop and validate a shortened version of the MDASI-UGI-Surg (the Short-MDASI-UGI-Surg), through time series clustering and symptom reduction strategies, that preserves the accuracy and sensitivity of the full instrument while substantially reducing respondent burden, thereby supporting its application in routine postoperative care.

## Methods

This study was conducted under a comprehensive protocol approved by the MD Anderson Institutional Review Board (Protocol #2021-0799). Development of the MDASI-UGI-Surg followed a three-phase process aligned with US Food and Drug Administration (FDA) guidance for PRO instrument development^17–19^. To achieve the third phase, prospective PRO collection (psychometric validation) was conducted using longitudinal data from patients undergoing UGI cancer surgery^10^. This paper focuses on quantifying longitudinal symptom trajectories and developing a shortened version of the instrument. All participants provided informed consent, and all procedures adhered to institutional ethical standards.

### Patients

Eligible participants were adults (age ≥ 18 years) who were able to speak and read English and were scheduled to undergo surgery for oesophageal, gastric, or pancreatic disease during October 2020 through September 2024. Patients were excluded if they had cognitive impairments that precluded reliable completion of the MDASI-UGI-Surg, required translation services, or were scheduled for multiorgan resections. Patients who did not complete the preoperative baseline survey, or whose operations were cancelled or aborted after baseline survey completion, were also excluded.

To increase sample size, patients were also included from a separate quality improvement project (Protocol #QIAB 527) focused on PRO collection after pancreatectomy and gastrectomy; the requirement for informed consent was waived for this protocol. In the quality improvement cohort, the MDASI for gastrointestinal cancer (MDASI-GI) instrument (previously validated in patients with gastrointestinal cancers^7^) was used. For both cohorts, patients who provided responses at a minimum of two timepoints were included in the analyses.

### MDASI, MDASI-GI, and MDASI-UGI-Surg

For analyses requiring a full-length reference instrument, a harmonized full MDASI instrument was constructed by integrating overlapping symptom and interference items from MDASI in patients with gastrointestinal cancer (MDASI-GI) and MDASI-UGI-Surg^10^. The MDASI^8^ is a validated 19-item tool for assessing cancer-related symptoms, independent of treatment or cancer type. It consists of 13 symptom items and six interference items, all rated on a numeric scale from 0 to 10, with 10 indicating the highest severity. The MDASI-GI extends the core MDASI with five additional symptom items (constipation, diarrhoea, difficulty swallowing, change in taste, and feeling bloated)^7^. The MDASI-UGI-Surg extends MDASI with eight UGI surgery-specific module items (difficulty swallowing, heartburn/reflux, diarrhoea, constipation, flushing/sweating, stomach feeling full, malaise, dizziness, and feeling cold).

For the combined analysis, overlapping items (difficulty swallowing, diarrhoea, constipation, and stomach feeling full) from the MDASI-GI were integrated with the MDASI-UGI-Surg to enable item-level harmonization across instruments. The MDASI-GI item feeling bloated was harmonized to stomach feeling full. Symptoms unique to the MDASI-UGI-Surg (reflux, flushing/sweating, dizziness) were retained for clustering analyses, because these were conducted using standardized time series scores for each symptom. To account for differences in the number of items across instruments, mean scores were calculated to minimize bias in validity assessments. Both symptom and interference items were scored on the same 0–10 numeric rating scale, ranging from ‘not present’ (0) to ‘as bad as you can imagine’ (10) for symptom items and from ‘did not interfere’ (0) to ‘interfered completely’ (10) for interference items. Given their shared scaling and continuous distribution, both domains were analysed using the same statistical framework to ensure consistency across items and to facilitate identification of representative items for the shortened instrument.

This harmonized full MDASI instrument was used for time series clustering and validation analyses. In contrast, the Short-MDASI-UGI-Surg was evaluated against this harmonized reference instrument.

### Data collection

In both study cohorts, study data were collected and managed using REDCap electronic data capture tools (Vanderbilt University, Nashville, TN, USA) hosted at The University of Texas MD Anderson Cancer Center^20,21^.

For both the MDASI-UGI-Surg validation study and the quality improvement project with the MDASI-GI, participants were sent e-mails before surgery and at 3, 7, 14, and 21 days, and 1, 3, and 6 months after surgery, with each e-mail including a secure link to the corresponding MDASI-UGI-Surg or MDASI-GI survey on REDCap. The link expired after 48 hours for the preoperative and 3-, 7-, 14-, and 21-day postoperative surveys. The 1-, 3-, and 6-month postoperative survey links expired after 2 weeks. In the MDASI-UGI-Surg validation study, the research coordinator proactively contacted patients via e-mail, telephone, and in-person visits to improve collection rates up to 1 month after surgery, whereas the MDASI-GI quality improvement project relied mostly on patients’ voluntary responses.

Disease- and treatment-specific information, including organ, pathological diagnosis, type of operation, and surgical approach (open, laparoscopic, or robotic), was collected by research personnel via manual review of electronic medical records.

### Statistical analysis

All correlation analyses were performed using timepoint-level aggregated mean symptom scores rather than individual patient-level data.

#### Time series clustering of postoperative symptom trajectories

Hierarchical clustering was performed to characterize longitudinal postoperative symptom trajectories using Ward’s minimum variance method with Euclidean distance on symptom-level standardized scores. Standardization was applied at the symptom level before clustering. To visualize cluster-averaged temporal patterns, additional *Z* score standardization across timepoints was applied solely for visualization purposes and was not used for cluster assignment, item reduction, or psychometric validation analyses. The number of clusters was determined based on dendrogram structure and clinical interpretability of symptom trajectories.

#### Item reduction

To minimize redundancy, multivariable correlation analyses were performed using symptom scores at the peak timepoint of each cluster. Items demonstrating high intercorrelation, defined by a Pearson correlation coefficient ≥ 0.55, were grouped. From each correlated group, representative symptoms were retained, selected based on clinical relevance, mean severity, and variance.

#### Validation

The psychometric performance of the shortened instrument was examined through three complementary approaches: concurrent validity, evaluated by the Pearson correlation coefficient (*r*) between mean scores obtained from the harmonized full MDASI instrument and Short-MDASI-UGI-Surg across all available timepoints; responsiveness, assessed by Pearson correlations between mean scores between the harmonized full MDASI instrument and the Short-MDASI-UGI-Surg at each perioperative timepoint; and known groups validity, tested by examining whether differences in mean item scores between patients with oesophageal, pancreatic, and gastric cancers measured by a Mann–Whitney *U* test at multiple postoperative time points were similarly observed in both the harmonized full MDASI instrument and the Short-MDASI-UGI-Surg.

#### Sensitivity analysis

As a sensitivity analysis, the analysis was restricted to MDASI-UGI-Surg respondents and whether the predefined acute, subacute, and persistent symptom clusters retained their characteristic temporal trajectories was examined. Cluster-level trajectories were visualized using standardized symptom scores calculated within each postoperative timepoint.

Statistical significance was defined as two-sided *P* < 0.05. All analyses were performed using JMP Pro version 18 (SAS Institute Inc., Cary, NC, USA).

## Results

### Study population

Of the 399 enrolled patients, 97 were excluded owing to incomplete preoperative assessments or less than two response timepoints, leaving 302 patients for analysis. Of these patients, 159 completed the MDASI-GI and 143 completed the MDASI-UGI-Surg. The most common primary tumour site was the pancreas (159, 52.6%), followed by the stomach (101, 33.4%) and oesophagus (42, 13.9%).

### Survey response rates

Survey completion rates varied by timepoint and questionnaire type. The MDASI-UGI-Surg cohort demonstrated consistently higher response rates. Across both instruments, response rates declined at later postoperative timepoints, with completion at postoperative months 3 and 6 averaging around 60%. Response rates are summarized by timepoint and instrument in *Table 1*.

**Table 1:** Summary of response rates by timepoint and instrument

	*n*	Preop	POD 3	POD 7	POD 14	POD 21	POM 1	POM 3	POM 6
**Total**									
*n*	302	302	259	221	224	245	256	186	183
%		100.0	85.8	73.2	74.2	81.1	84.8	61.6	60.6
**MDASI-UGI-Surg**									
*n*	143	143	137	139	132	132	137	90	92
%		100.0	95.8	97.2	92.3	92.3	95.8	62	64
**MDASI-GI**									
*n*	159	159	122	82	92	113	119	96	91
%		100.0	76.7	51.6	57.9	71.1	74.8	60	57

Data show the number of patients responding at each timepoint, with percentages calculated based on the total number of patients at that timepoint. Preop, before surgery; POD, postoperative day; POM, postoperative month; MDASI-UGI-Surg, MD Anderson Symptom Inventory for upper gastrointestinal surgery; MDASI-GI, MD Anderson Symptom Inventory for gastrointestinal cancer.

To assess potential attrition bias, baseline characteristics were compared between responders and non-responders at postoperative month 3. There were no significant differences in age, PRO instrument, or baseline mean symptom scores between groups. Disease distribution differed between groups, which may reflect differences in postoperative course, follow-up requirements, and ongoing oncological treatment across disease types (*Table S1*).

### Time series clustering of symptom trajectories

**Fig. 1:**
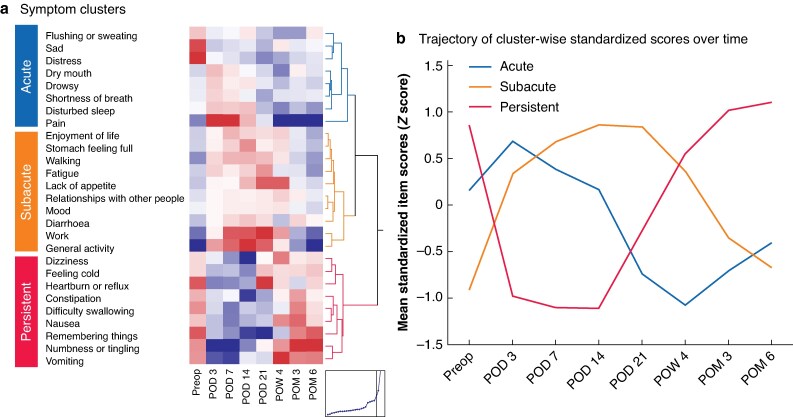
Time series clustering of postoperative symptom trajectories The 27 symptom/interference items were derived from a harmonized full MDASI instrument that integrates MDASI-UGI-Surg and MDASI-GI items (see Methods). The 27 symptoms were evaluated at eight perioperative time points and clustered according to their longitudinal trajectory patterns using time-series clustering. **a** Three distinct symptom clusters were identified: acute symptoms, which peaked on POD 3 and rapidly declined; subacute symptoms, which peaked on POD 21; and persistent symptoms, which remained elevated through POM 6. **b** Standardized mean scores for each cluster are shown over time. MDASI, MD Anderson Symptom Inventory; MDASI-UGI-Surg, MD Anderson Symptom Inventory for upper gastrointestinal surgery; MDASI-GI, MD Anderson Symptom Inventory for gastrointestinal cancer; POD, postoperative day; POM, postoperative month; Preop, before surgery; POW, postoperative week.

Time series clustering analysis of 27 symptom/interference trajectories across eight perioperative timepoints in all 302 patients revealed three distinct clusters, differentiated by their temporal patterns and relative peak (*Fig. 1*). In the acute cluster (eight symptoms), symptoms peaked sharply on postoperative day (POD) 3 and declined quickly thereafter, reflecting an acute postoperative burden. The symptoms in the acute cluster were dry mouth, drowsiness, shortness of breath, disturbed sleep, flushing or sweating, sadness, distress, and pain. In the subacute cluster (ten symptoms), symptoms had a relatively low preoperative burden, with a delayed relative peak at POD 21, and gradual resolution thereafter. The subacute symptoms included enjoyment of life, stomach feeling full, walking, fatigue, relationships with other people, mood, lack of appetite, diarrhoea, working, and general activity. In the persistent cluster (nine symptoms), symptoms in this cluster were characterized by a sustained burden from the preoperative period through 6 months after surgery, with relatively little evidence of improvement over time. The persistent symptoms consisted of dizziness, feeling cold, constipation, difficulty swallowing, nausea, problems remembering things, heartburn or reflux, numbness or tingling, and vomiting.

### Cluster-specific item reduction

**Fig. 2:**
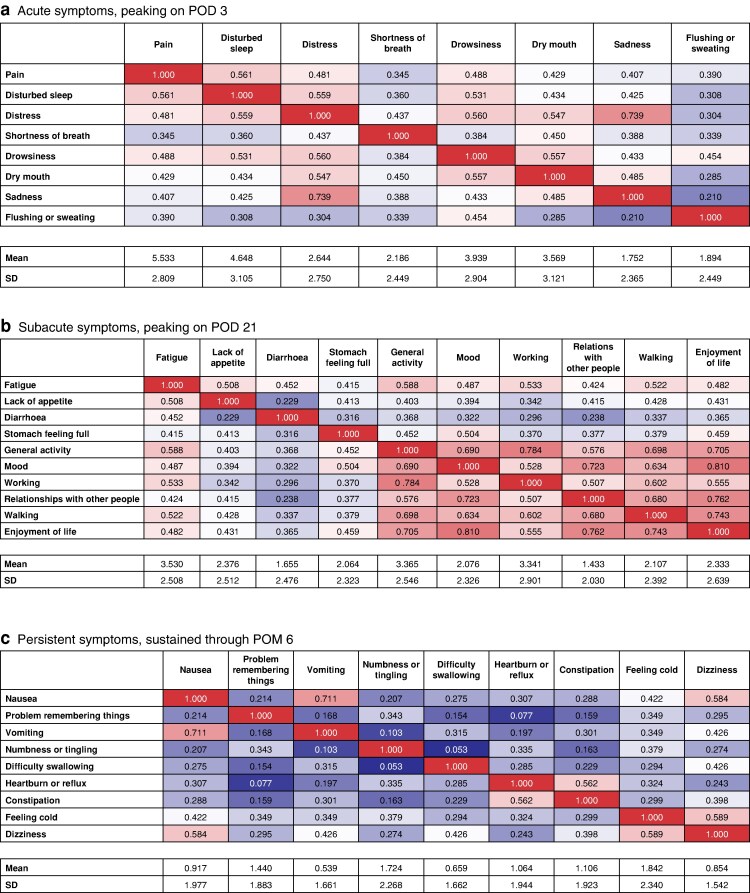
Cluster-specific correlation analysis and symptom reduction Pearson correlation coefficients, *r*, are shown unless otherwise indicated. **a** In the acute symptom cluster, strong intersymptom correlations were observed between disturbed sleep and pain and between distress and disturbed sleep, drowsiness, and sadness. Shortness of breath appeared relatively independent. Based on clinical relevance and statistical criteria, three representative symptoms, namely pain, distress, and shortness of breath, were selected. **b** In the subacute cluster, fatigue was strongly correlated with general activity, whereas enjoyment of life showed broad associations with mood, walking, relationships with other people, and working. Lack of appetite, diarrhoea, and stomach feeling full were more independent. Six representative symptoms were selected. **c** In the persistent cluster, nausea showed strong correlations with vomiting and dizziness, whereas problem remembering things, numbness or tingling, and heartburn or reflux were more independent. Difficulty swallowing and constipation were excluded due to low severity scores, and feeling cold was excluded due to low specificity. Four representative symptoms were selected. These 13 symptoms were compiled to construct the short version of the MD Anderson Symptom Inventory for upper gastrointestinal surgery. Cell shading represents mean symptom severity. Red indicates higher severity scores, and blue indicates lower severity scores. POD, postoperative day; POM, postoperative month; SD, standard deviation.

In the acute symptom cluster (*Fig. 2a*), multivariable correlation analysis demonstrated several notable associations. Disturbed sleep was strongly correlated with pain, and disturbed sleep, drowsiness, and sadness were all highly correlated with distress. In addition, drowsiness was strongly correlated with dry mouth, whereas shortness of breath appeared to be relatively independent from the other symptoms within this cluster. Notably, flushing or sweating exhibited a particularly low mean severity score (1.89), suggesting limited clinical relevance. Based on these findings, three symptoms (pain, distress, and shortness of breath) were selected to represent this cluster.

In the subacute symptom cluster (*Fig. 2b*), fatigue was strongly correlated with general activity, whereas enjoyment of life was broadly correlated with walking, relationships with other people, working, mood, and general activity. In contrast, lack of appetite, diarrhoea, and stomach feeling full were relatively independent. Six symptoms were selected to best characterize this cluster: general activity, fatigue, lack of appetite, diarrhoea, stomach feeling full, and enjoyment of life.

In the persistent symptom cluster (*Fig. 2c*), weak interitem correlations were observed overall. However, nausea was strongly correlated vomiting and was moderately associated with dizziness. In contrast, problems remembering things, numbness or tingling, and heartburn or reflux appeared largely independent. Although difficulty swallowing and constipation were also independent, their mean severity scores were low (0.7 and 1.1, respectively). Feeling cold was excluded due to its non-specific nature. Accordingly, four key symptoms commonly reported after UGI operations and during cancer treatment (that is, nausea, problems remembering things, numbness or tingling, and heartburn or reflux) were retained to represent this group.

Based on these findings and careful discussions among experts, a 13-item short survey was developed based on MDASI-UGI-Surg, named the Short-MDASI-UGI-Surg (*Table 2*). To preserve the conceptual structure of the original MDASI framework, the survey format of the Short-MDASI-UGI-Surg was maintained with separate sections for symptom severity and symptom interference, as shown in *Fig. S1*.

**Table 2:** Items included in the MDASI-UGI-Surg and Short-MDASI-UGI-Surg

MDASI-UGI-Surg (27 items)	Classification	Short-MDASI-UGI-Surg (13 items)
**Acute symptoms**		**Acute symptoms**
Pain	Core symptoms	Pain
Disturbed sleep	Core symptoms	
Distress	Core symptoms	Distress
Shortness of breath	Core symptoms	Shortness of breath
Drowsiness	Core symptoms	
Dry mouth	Core symptoms	
Sadness	Core symptoms	
Flushing or sweating*	Module symptoms	
**Subacute symptoms**		**Subacute symptoms**
Fatigue	Core symptoms	Fatigue
Lack of appetite	Core symptoms	Lack of appetite
General activity	Interference items	General activity
Mood	Interference items	
Working (including work around the house)	Interference items	
Relationships with other people	Interference items	
Walking	Interference items	
Enjoyment of life	Interference items	Enjoyment of life
Diarrhoea	Module symptoms	Diarrhoea
Stomach feeling full^†^	Module symptoms	Stomach feeling full
**Persistent symptoms**		**Persistent symptoms**
Nausea	Core symptoms	Nausea
Problem remembering things	Core symptoms	Problem remembering things
Vomiting	Core symptoms	
Numbness or tingling	Core symptoms	Numbness or tingling
Difficulty swallowing	Module symptoms	
Heartburn or reflux	Module symptoms	Heartburn or reflux
Constipation	Module symptoms	
Feeling cold	Module symptoms	
Dizziness	Module symptoms	

Questions are asked using simple and consistent language, such as ‘How severe are your symptoms (scale 0–10) when your [symptom/item] is at its worst?’ Patients are asked to recall the severity of their symptoms over the past 24 hours. *The MDASI-UGI-Surg item Flushing or sweating was aligned with the Flushing item in the MDASI-GI questionnaire. †The MDASI-UGI-Surg item Stomach full was aligned with the Bloated item in the MDASI-GI for comparative purposes. MDASI-UGI-Surg, MD Anderson Symptom Inventory for upper gastrointestinal surgery; Short-MDASI-UGI-Surg, short version of the MDASI-UGI-Surg; MDASI-GI, MD Anderson Symptom Inventory for gastrointestinal cancer.

### Validation of the Short-MDASI-UGI-Surg

To validate the performance of the Short-MDASI-UGI-Surg, three complementary analyses were conducted.

#### Concurrent validity

**Fig. 3:**
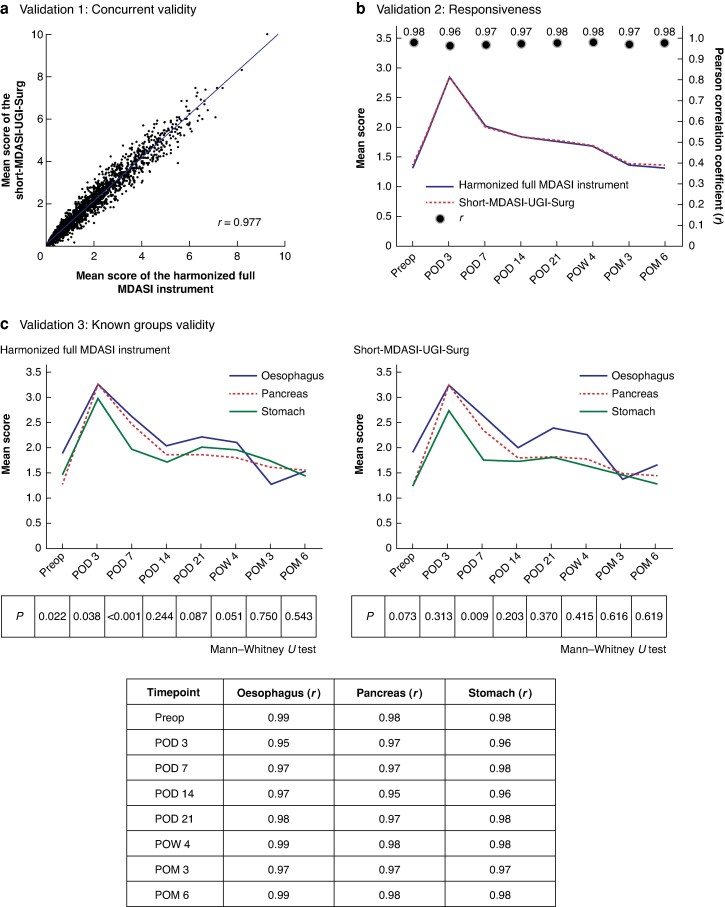
Validation of the Short-MDASI-UGI-Surg **a** Concurrent validity. Mean scores of the Short-MDASI-UGI-Surg were strongly correlated with those of the harmonized full MDASI instrument across all timepoints (*r* = 0.98). **b** Responsiveness. Time series symptom trends remained highly concordant between the Short-MDASI-UGI-Surg and the harmonized full MDASI instrument (*r* > 0.96). **c** Known groups validity. Symptom trajectories stratified by tumour site (oesophageal, pancreatic, gastric cancer) showed similar patterns and high correlation (*r* > 0.96 across all groups and timepoints), confirming the ability of the Short-MDASI-UGI-Surg to reproduce clinically meaningful distinctions. The lower panel shows tumour-specific correlations between the harmonized full MDASI instrument and the Short-MDASI-UGI-Surg at each postoperative timepoint, serving as a subgroup analysis of responsiveness rather than a formal known groups validity assessment. This provided further supportive evidence for responsiveness (oesophagus: *r* = 0.95–0.99; pancreas: *r* = 0.95–0.98; stomach: *r* = 0.96–0.98). Short-MDASI-UGI-Surg, short version of the MD Anderson Symptom Inventory for upper gastrointestinal surgery; MDASI, MD Anderson Symptom Inventory; POD, postoperative day; POM, postoperative month; Preop, before surgery; POW, postoperative week.

The correlation between mean scores calculated from the harmonized full MDASI instrument and the Short-MDASI-UGI-Surg across all available time points was examined. A strong linear relationship was observed (*r* = 0.98), indicating that the Short-MDASI-UGI-Surg reliably reflects the overall symptom burden assessed by the harmonized full MDASI instrument (*Fig. 3a*).

#### Responsiveness

Mean scores at each timepoint were compared between the harmonized full MDASI instrument and the Short-MDASI-UGI-Surg. Time series profiles were highly concordant across all time points (*r* > 0.96), demonstrating that the Short-MDASI-UGI-Surg retained the responsiveness of the harmonized full MDASI instrument to changes in symptom burden over time (*Fig. 3b*).

#### Known groups validity

Known groups validity was supported by differences in symptom scores between oesophageal, pancreatic, and gastric cancer groups across several postoperative time points. On POD 3, both the harmonized full MDASI instrument and Short-MDASI-UGI-Surg showed clear differences (*P* = 0.038 and *P* = 0.022, respectively). These group differences remained consistent across later timepoints, such as POD 7 (*P* < 0.001 *versus P* < 0.001) and POD 21 (*P* = 0.083 *versus P* = 0.074), indicating that the Short-MDASI-UGI-Surg preserved the discriminative validity of the harmonized full MDASI instrument. In addition, *Fig. 3c* shows the correlation coefficients between the mean scores of the harmonized full MDASI instrument and the Short-MDASI-UGI-Surg for the oesophageal, pancreatic, and gastric cancer groups across multiple postoperative timepoints. This provided further supportive evidence for responsiveness (oesophagus: *r* = 0.95–0.99; pancreas: *r* = 0.95–0.98; stomach: *r* = 0.96–0.98).

### Sensitivity analysis

To assess the robustness of the short-form development strategy to instrument source, a sensitivity analysis restricted to participants who completed the MDASI-UGI-Surg instrument only was conducted. The standardized cluster-wise temporal trajectories of the acute, subacute, and persistent symptom clusters remained highly consistent with those observed in the primary analysis (*Fig. S2*).

## Discussion

In this study, the Short-MDASI-UGI-Surg was developed, condensed from the MDASI-UGI-Surg, designed for longitudinal symptom monitoring after UGI cancer surgery. Despite halving the number of symptom items, the Short-MDASI-UGI-Surg demonstrated strong performance, including high internal consistency, excellent agreement with the harmonized full MDASI instrument, and robust reproducibility. Importantly, the reduced instrument successfully retained the temporal structure of symptom burden, preserving core patterns across the acute, subacute, and persistent phases of postoperative recovery. In parallel, the clustering analysis enabled time series annotation of the original symptom set, offering additional insights that may inform targeted interventions. These findings highlight the potential utility of the Short-MDASI-UGI-Surg for simplifying symptom monitoring and enabling timely, data-driven clinical decision-making.

The Short-MDASI-UGI-Surg embodies several paradigm-shifting strengths. Its concise, single-page format enables completion in approximately 1 minute, making it feasible for clinical practice. Further, it was deliberately designed to capture symptoms most relevant to UGI surgery, rigorously guided by cluster analyses of robust longitudinal data. This methodological foundation distinguishes the Short-MDASI-UGI-Surg from previous attempts to reduce questionnaire burden. Existing short-form PRO instruments were primarily designed for cross-sectional assessment^22–25^, providing limited resolution for perioperative symptom trajectories. Consequently, there has been no concise, surgery-specific instrument capable of capturing the dynamic evolution of postoperative recovery. The Short-MDASI-UGI-Surg was developed to address this gap, enabling efficient longitudinal monitoring of symptom burden while maintaining conceptual continuity with the original MDASI framework.

In this study, the clustering of symptoms by time-dependent relative peaks represents the first systematic attempt to categorize postoperative symptoms according to their trajectory type. This structure carries immediate and transformative clinical relevance: interventions can be designed according to biologically and temporally coherent domains, enabling more precise, timely, and effective symptom management.

Acute cluster symptoms, namely pain, distress, and shortness of breath, represent the immediate physiological and emotional impact of surgery and opioid use. These require rapid, targeted intervention. The acute symptom cluster likely reflects general postoperative phenomena common to major surgery, including perioperative fasting and analgesic effects, rather than only UGI-specific mechanisms. Standardized perioperative strategies such as opioid-sparing analgesia^26^, respiratory care^27^, psychological support^28^, and enhanced recovery after surgery protocols^29–31^ improve short-term recovery and should be systematically studied against acute symptom trajectories. The growing adoption of minimally invasive approaches, including robotic surgery, may further reduce acute symptom burden^32–34^, but this hypothesis demands rigorous prospective evaluation. The Short-MDASI-UGI-Surg may serve as a practical foundation for future studies designed to evaluate the impact of perioperative care pathways, including enhanced recovery after surgery protocols and postoperative complications, on longitudinal PROs.

Subacute cluster symptoms, for example fatigue, appetite loss, diarrhoea, and reduced activity, emerge after the acute phase and persist during the vulnerable weeks of recovery. They reflect altered gastrointestinal physiology, loss of organ function, and nutritional deficits. Targeted interventions such as early dietician involvement, nutritional and enzyme supplementation, and tailored dietary modifications can mitigate digestive symptoms, whereas structured rehabilitation programmes reduce fatigue and restore activity^35^. Notably, the enjoyment of life item may serve as a sentinel marker of overall recovery, highlighting the need for multidisciplinary care that integrates nutrition, rehabilitation, and psycho-oncology.

Persistent cluster symptoms, namely neuropathy, reflux, nausea, and cognitive complaints, extend into survivorship and represent long-term treatment effects. Though often less intense, their chronicity imposes a substantial long-term quality-of-life burden. Neuropathic symptoms may be alleviated by duloxetine or gabapentin with supportive therapy^36^. Reflux can be managed with lifestyle modification and long-term proton pump inhibitors^37^. Cognitive complaints (‘chemo brain’) require proactive strategies such as cognitive rehabilitation, sleep hygiene, and neuropsychological support^38,39^. The early identification and monitoring of persistent symptoms enable modification of the chemotherapy regimen, timely specialist referral, and individualized survivorship planning.

Although symptom and interference items were analysed together on a unified 0–10 numeric scale to ensure consistent statistical evaluation, the final Short-MDASI-UGI-Surg maintained the conceptual integrity of the original MDASI framework by separating these domains in the survey format. This approach ensures conceptual clarity while preserving analytical comparability across domains. The finalized short form, shown in *Fig. S1*, adheres to the established MDASI structure, which enable seamless integration into electronic platforms. Future qualitative interview studies will further assess the clarity and relevance of both domains and refine the content validity of the instrument. Moving forward, the plan is to implement the Short-MDASI-UGI-Surg into the electronic medical record at The University of Texas MD Anderson Cancer Center. Its sensitivity, brevity, and ease of integration make it ideal for routine use. Embedding the short form will enable real-time, prospective symptom monitoring with minimal burden, allowing frequent assessments in both clinic and inpatient settings. Routine use will provide efficient, comprehensive evaluation of symptom burden, directly supporting patient-centred care and improving satisfaction with surgical and cancer care. In parallel, the aim is to translate the Short-MDASI-UGI-Surg into multiple languages to enable international use, cross-cultural validation, and broader incorporation of PROs into clinical studies. It is anticipated that the outcomes of these implementation efforts will be reported soon, advancing global integration of patient-reported symptom monitoring in perioperative oncology.

Several study limitations should be acknowledged. First, to enrich symptom data, data sets were combined from both the MDASI-UGI-Surg and MDASI-GI instruments. Although this approach was necessary to ensure adequate sample size and timepoint coverage (the latter collected under a quality improvement protocol in pancreatic and gastric cancer patients, and the former under a more controlled prospective study), it introduced heterogeneity. Importantly, the cluster-wise temporal trajectories were preserved when analyses were restricted to MDASI-UGI-Surg respondents only (*Fig. S2*). Although symptom scores were standardized and clustering was based on mean trajectories across individuals, thereby minimizing bias introduced by instrument differences, this heterogeneity may nonetheless influence interpretability. Second, the response rates at later timepoints (that is, postoperative months 3 and 6) were suboptimal, reflecting the practical challenges of intensive longitudinal collection of PRO data in surgical patients. Finally, a key limitation of the study was the absence of qualitative patient input to confirm content validity. According to PRO development standards, cognitive interviews with patients are required to ensure that all items most important to patients are retained. Therefore, future research should incorporate patient interviewing across different postoperative phases to establish content validity. This work represents an initial investigation, acknowledging that content validity ultimately requires direct patient input, which will be pursued in future qualitative studies before finalizing the PRO instrument. Accordingly, the Short-MDASI-UGI-Surg should be considered a provisional instrument pending formal qualitative validation through patient cognitive interviews, as recommended by FDA PRO guidance^17^. Although the item reduction strategy prioritized longitudinal relevance and severity, exclusion of infrequent symptoms may carry a risk of underrepresenting clinically important issues for specific patients. In addition, some excluded symptoms demonstrated consistently low mean severity across timepoints, which may partly reflect floor effects rather than the absence of clinical relevance. Ongoing qualitative studies will be essential to confirm content validity and refine item selection before widespread clinical implementation. In addition, this study was conducted at a single, high-volume cancer centre with extensive perioperative resources, which may limit generalizability to lower-volume or resource-limited settings. In addition, detailed information on surgical approach, disease stage, and perioperative oncological treatments was not uniformly available, precluding treatment-specific subgroup analyses; future multicentre studies will be needed to evaluate how these factors influence symptom trajectories and clinical implementation. Accordingly, external validation in multicentre and international cohorts will be essential before widespread clinical adoption.

In summary, the 13-item short survey based on the MDASI-UGI-Surg retains the essential temporal dynamics of postoperative symptom recovery while significantly reducing patient and researcher burden. The Short-MDASI-UGI-Surg demonstrated strong psychometric performance and preserved symptom trajectory patterns across acute, subacute, and persistent phases. This tool offers a practical, efficient solution for longitudinal symptom monitoring in surgical oncology and holds promise for facilitating more timely, patient-centred interventions. Future prospective, multicentre validation is warranted to confirm its feasibility, responsiveness, and impact on postoperative care quality.

## Supplementary Material

zrag026_Supplementary_Data

## Data Availability

The data sets generated and analysed during the present study are available from the corresponding author upon reasonable request.
