# Supplementary material for: Development and validation of a short version of the MD Anderson Symptom Inventory for upper gastrointestinal surgery (short-MDASI-UGI-Surg) for postoperative patient-reported outcome-based care
Source: BJS Open. 2026 Apr 15;10(2):zrag026. doi: 10.1093/bjsopen/zrag026 (PMC13080355; doi:10.1093/bjsopen/zrag026)
Supplement: zrag026_Supplementary_Data [file zrag026_supplementary_data.docx]

**Development and Validation of a Short Version of the MD Anderson Symptom Inventory for Upper Gastrointestinal Surgery (Short-MDASI-UGI-Surg) for Postoperative Patient-Reported Outcome–Based Care**

Taisuke Imamura, MD, PhD^a^, Koichi Tomita, MD, PhD^a^, Paula Marincola Smith, MD, PhD^b^, Maho Takayama, MD^a^, Anneliese Hierl, MD^a^, Xin Shelley Wang, MD, MPH^c^, Loretta A. Williams, PhD^c^, Kyle G. Mitchell, MD^d^ , Ravi Rajaram, MD, MSc^d^, David Rice, MD^d^, Wayne Hofstetter, MD^d^, Mara B. Antonoff, MD^d^, Reza Mehran, MD, MSc^d^, Ara Vaporciyan, MD^d^, Garrett Walsh, MD^d^, Jessica E. Maxwell, MD, MBA^a^, Rebecca A. Snyder, MD, MPH^a^, Michael P. Kim, MD^a^, Ching-Wei D. Tzeng, MD^a^, Paul Mansfield, MD^a^, Stephen Swisher, MD^d^, Jeffrey E. Lee, MD^a^, Brian D. Badgwell, MD^a^, Matthew H. G. Katz, MD^a^, Naruhiko Ikoma, MD, MS^a^

^a^ *Department of Surgical Oncology, The University of Texas MD Anderson Cancer Center, Houston, TX*

^b^ *Department of Department of Colon & Rectal Surgery, The University of Texas MD Anderson Cancer Center, Houston, TX*

^c^ *Department of Symptom Research, The University of Texas MD Anderson Cancer Center, Houston, TX*

^d^ *Department of Thoracic and Cardiovascular Surgery, The University of Texas MD Anderson Cancer Center, Houston, TX*

**Corresponding Author:** Naruhiko Ikoma, MD, MS

Department of Surgical Oncology, The University of Texas MD Anderson Cancer Center

1400 Pressler Street, Houston, TX 77030

Phone: 713-563-2354

Email: [nikoma@mdanderson.org](mailto:nikoma@mdanderson.org)

**Supplementary Materials - Index**

| **Supplementary Figures and Tables** |  |
| --- | --- |
| Supplementary Figure 1 | *page 3* |
| Supplementary Figure 2 | *page 4* |
| Supplementary Table 1 | *page 5* |

**Supplementary Figure 1. Short-MDASI-UGI-Surg questionnaire**

The Short-MDASI-UGI-Surg is a **13-item patient-reported outcome instrument derived from the MDASI-UGI-Surg**, developed to assess postoperative symptom burden in patients undergoing upper gastrointestinal surgery.

Supplementary Figure 2. Sensitivity analysis restricted to MDASI-UGI-Surg respondents

**
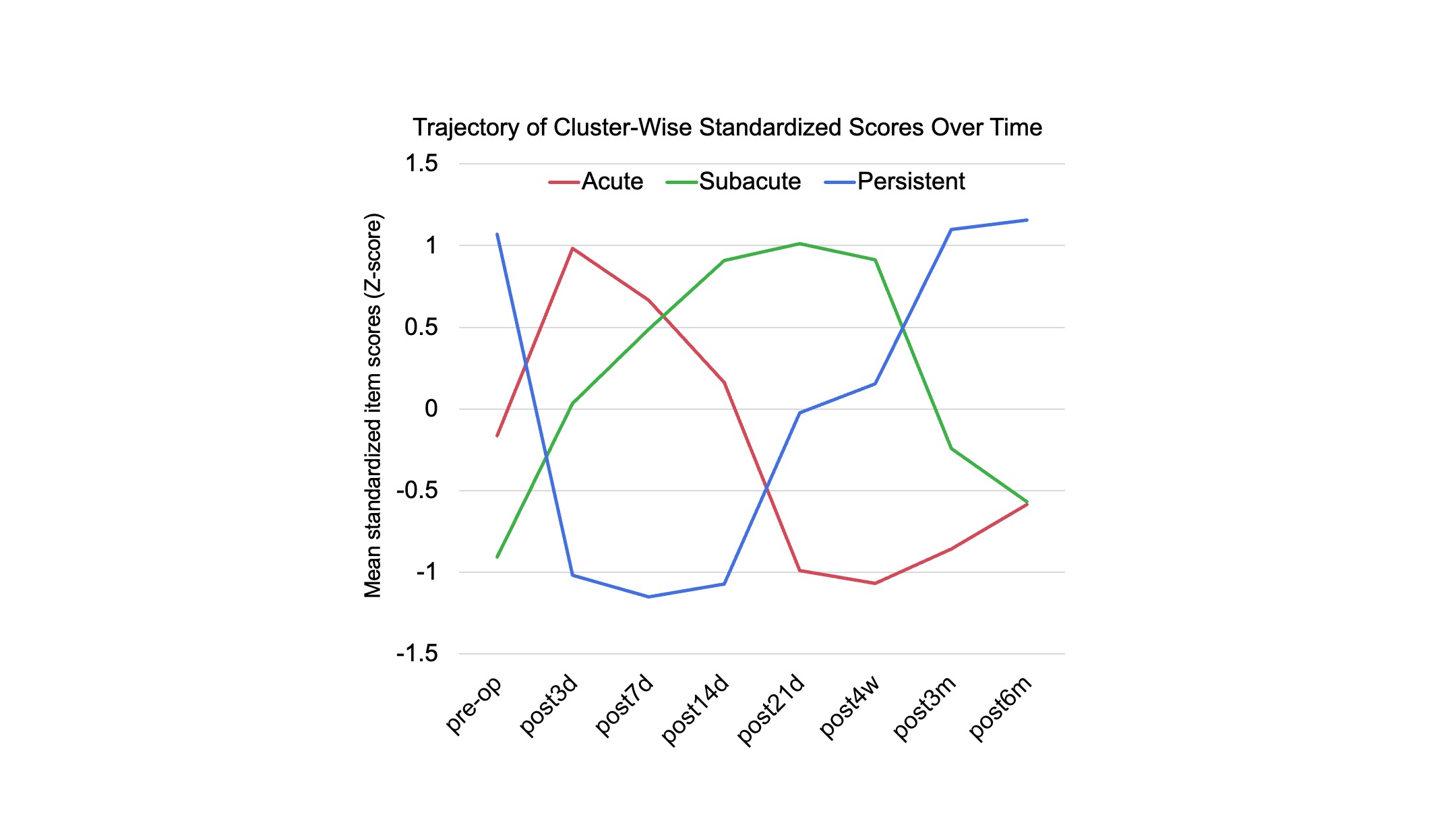
**

Cluster-wise temporal symptom trajectories remained consistent when analyses were restricted to patients who completed the MDASI-UGI-Surg only, supporting the robustness of the clustering strategy.

Supplementary Table 1.

| **Supplementary Table 1**. Comparison of baseline characteristics between responders and non-responders at postoperative month 3 | | | | |
| --- | --- | --- | --- | --- |
|  |  | Responder | Non-responder |  |
|  |  | 186 | 116 | *P* |
| Instrument | MDASI-UGI-Surg | 90 | 53 | 0.757 |
|  | MDASI-GI | 96 | 63 |  |
|  |  |  |  |  |
| Disease | Esophagus | 24 | 18 | 0.013 |
|  | Stomach | 112 | 48 |  |
|  | Pancreas | 53 | 47 |  |
|  |  |  |  |  |
| Age |  | 64 (56–73) | 61 (48–73) | 0.155 |
|  |  |  |  |  |
| Baseline symptom score | (mean at pre-op) | 0.87 (0.39–1.57) | 1 (0.26–2.45) | 0.389 |
|  |  |  |  |  |
